# Lessons learned on access, equity, and collaboration between public hospital Infectious Diseases leadership and the Health Department during the 2025 Legionnaires’disease outbreak response in New York City

**DOI:** 10.1017/ash.2026.10812

**Published:** 2026-08-28

**Authors:** Judith Ratcliffe, Kelsey Lamb, Madhuri Martin, Justin Chan, Emily McGibbon, Tristan McPherson, Briana Episcopia, Vikramjit Mukherjee, Andrew Wallach, Sharon Mannheimer, Ofole Mgbako

**Affiliations:** 1 NYU Grossman School of Medicine, New York, NY, USA; 2 NYC Health + Hospitals/Bellevue, New York, NY, USA; 3 NYC Department of Health and Mental Hygiene, New York, NY, USA; 4 NYC Health + Hospitals/Kings County, Brooklyn, NY, USA; 5 NYC Health + Hospitals/Harlem, New York, NY, USA

## Abstract

This commentary presents lessons learned and unique aspects of the public health response of infectious disease physicians, infection prevention and control specialists, and the New York City Health Department to the 2025 *Legionella* outbreak in Harlem.

## Background

**Figure 1.**
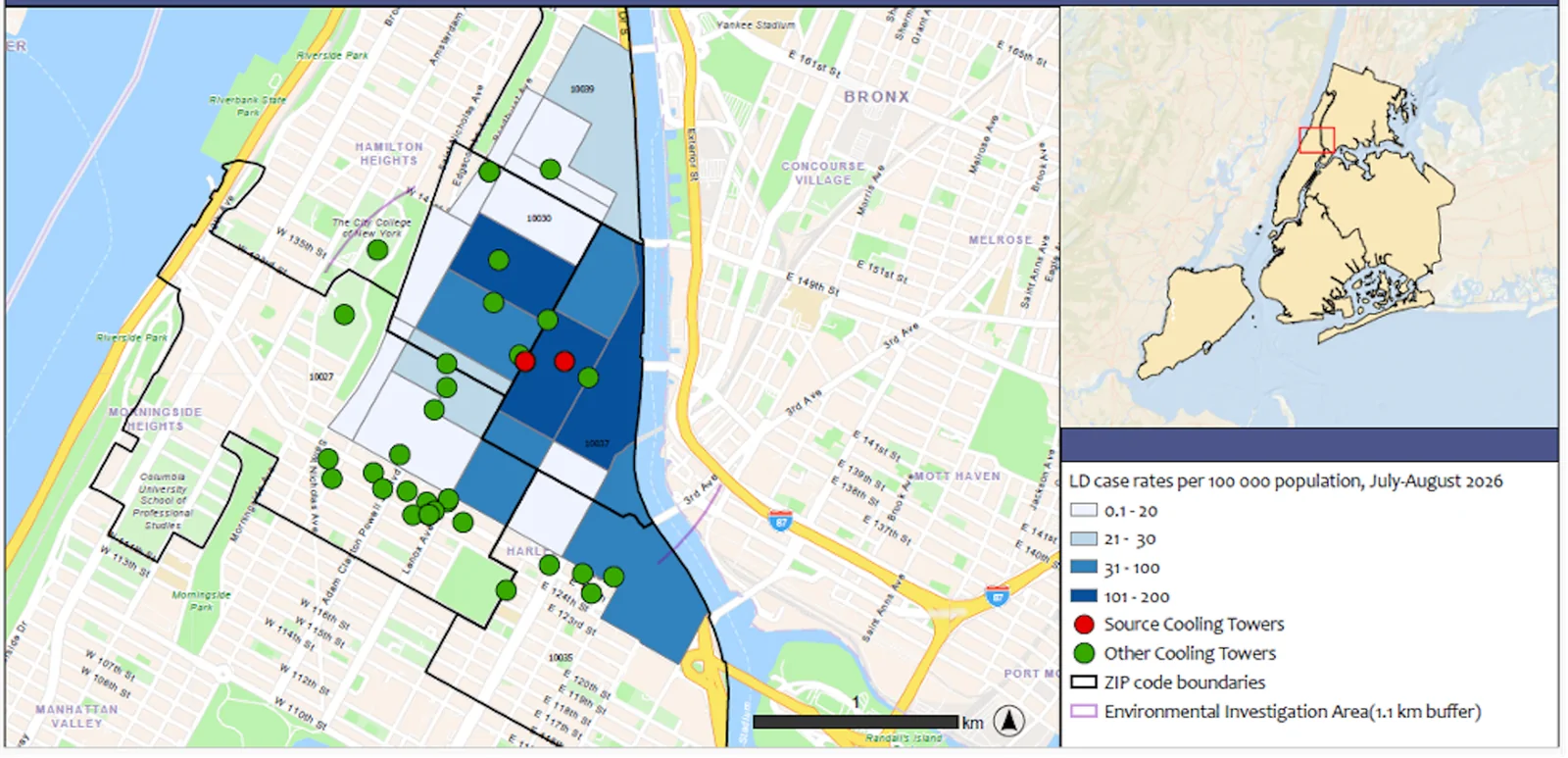
Figure 1 long description. Map of the cooling tower systems in the Harlem *Legionella* outbreak zone and confirmed outbreak cases by census tract, July – August 2025.

The 2025 Legionnaires’ disease (LD) outbreak in New York City (NYC) was the city’s largest LD outbreak since 2015, leading to 118 confirmed cases and 7 deaths.^1^ The NYC Health Department first detected a new cluster of LD cases on July 25, 2025, affecting 5 zip codes within Central Harlem, a predominantly Black community in NYC. After extensive investigation and environmental testing, *Legionella pneumophila* from patient specimens was identified by whole genome sequencing (WGS) and matched to two cooling towers, one at NYC Health+Hospitals (H+H)/Harlem and another at a nearby construction site (Figure 1).^1^ NYC Health+Hospitals (H+H) is the largest municipal health care system in the United States, providing healthcare services for over 1 million individuals across 11 acute care hospitals.

*Legionella*, the bacteria that causes LD, colonizes man-made water systems such as cooling towers and can persist within protozoa or in biofilms.^2^ Factors that promote bacterial growth include warm water temperature (25°C to 42°C), stagnation, and environmental factors such as aging water infrastructure and inadequate maintenance, which often impacts communities with lack of infrastructure investment or with high levels of poverty.^2,3^ LD is a disease that particularly impacts medically vulnerable populations including individuals over the age of 50, those with immunocompromising conditions such as hematologic malignancy, and individuals with tobacco use disorder.^4^ Typical signs and symptoms of LD include dyspnea, cough, and fever; more severe manifestations can include pneumonia, renal failure, rhabdomyolysis, cardiac disease such as myopericarditis, and even encephalopathy.^5,6^

In the past 10 years, there have been several LD outbreaks in NYC linked to cooling towers, disproportionately affecting marginalized communities with high percentages of racial/ethnic minorities. In 2015, 138 cases were identified in the South Bronx linked to a cooling tower, with 128 hospitalizations and 16 deaths.^7,8^ In 2022, also in the South Bronx, 30 cases were identified with 2 patient deaths.^9^ Following the 2015 outbreak, Local Law 77 was passed, mandating the registration, maintenance and certification of cooling towers throughout NYC.^10^

Prior LD outbreaks have laid bare the need for close collaboration between local health departments, hospital epidemiologists, and infectious diseases (ID) clinicians to enable prompt and thorough case identification and collection of clinical specimens for effective outbreak investigation and control. During the 2025 Central Harlem outbreak, most patients initially diagnosed with LD were admitted to NYC H+H/Harlem, with some severe cases transferred to other NYC H+H network hospitals to provide load balancing across the NYC H+H system. In doing so, this improved access and capacity at NYC H+H/Harlem to accept additional admissions in support of the outbreak response. Ninety-two individuals were hospitalized in the outbreak with 24 (26%) requiring intensive care, demonstrating the high risk of severe disease with LD.^11^

NYC Health Department leadership, NYC H+H/Bellevue ID providers and infection prevention and control (IP&C) specialists worked together to leverage hospital surveillance systems and educate front-line providers about early identification of LD and intervention for people at increased risk of severe disease. This commentary seeks to review lessons learned from the coordination of the ID service and the IP&C department within NYC H+H/Bellevue, along with the NYC Health Department, to increase access to care and work toward an equitable outbreak response. Effective communication was vital and may serve as an important model for future outbreak response strategies.

## Coordination of outbreak response

The NYC Health Department was first alerted to a potential LD cluster on July 25, 2025 via a spatiotemporal analysis signal in their surveillance system, whereby infectious disease incident data are evaluated daily and an alert is generated whenever there is a cluster of cases in space and time that would be highly unlikely to occur by chance.^12^ These alerts trigger investigation of a potential outbreak. On the date of cluster detection, the NYC Health Department received 8 reports of positive *Legionella* urine antigen tests (UAT) from patients at NYC H+H/Harlem.

The NYC Health Department team directly reached out to the ID Division Chief and Infection Prevention & Control (IP&C) at H+H/Harlem, the H+H System IP&C director, the H+H Ambulatory Care Chief Medical Officer (CMO), and the H+H Virtual Express Care Director to support mobilization of additional resources within a couple of days of outbreak detection. The NYC Health Department then met with H+H/Harlem Medicine Leadership, including the CMO and division heads for Internal Medicine and Emergency Medicine, as well as the NYC H+H Chief Biopreparedness Officer. The NYC Health Department communicated with the H+H Central Office who were in contact with division chiefs who could send internal messaging about LD throughout the hospital system.^13^ The H+H/Bellevue IP&C department and the NYC Department of Health corresponded several times a week during the outbreak.

NYC H+H systemwide IP&C leadership provided just-in-time training materials including information regarding the cluster of cases in the affected zip codes, recommended diagnostic approaches and testing guidance, and shared updated treatment guidance to support clinicians actively seeing patients. Information on outbreak case counts and public health recommendations was provided through alerts to the H+H system ID Clinical Council, Internal Medicine Clinical Council, Emergency Medicine Clinical Council, and Ambulatory leadership. These Councils were asked to amplify the information and recommendations to all frontline staff in their respective departments.

## Early diagnostic testing challenges with sputum culture collection

The IP&C department developed a systemwide syndromic surveillance tool—a database that could filter cases based on patient demographics, zip codes, and hospital unit locations. This surveillance dashboard was located within Electronic Health Record (EHR) and allowed the IP&C department to provide updated daily case counts and to follow the epidemic curve within the H+H hospital system. Targeted surveillance was also implemented for all patients admitted with a diagnostic code of community-acquired pneumonia from the five zip codes involved in the outbreak; this served as the case definition used to prompt testing within H+H. Upon identifying suspected LD cases through these surveillance tools, primary inpatient clinical teams were reminded to order a *Legionella* UAT, dedicated *Legionella* respiratory culture, and PCR prior to or soon after starting antibiotics for suspected LD through a real-time chat function in EHR. Respiratory culture is the gold standard for the NYC Health Department, which can then perform WGS on isolates, compare them to isolates from environmental testing, and identify the outbreak source. Notably, IP&C routinely submits a report of positive *Legionella* test results directly to the NYC Health Department.

IP&C goals were threefold: first, to improve culture yield by testing as soon as possible after presentation, ideally prior to antibiotic administration; second, to identify the proportion of community-acquired pneumonia cases caused by *Legionella* to initiate treatment early; and third, to support the determination of whether isolates from sputum culture matched environmental specimens obtained from the community. This active case-finding approach, along with coordination with clinicians and the health department, allowed the IP&C department to improve efforts to send appropriate diagnostic testing for suspected cases.

While communication was sent to frontline teams on the importance of collecting sputum culture specimens, we learned these proved very difficult to collect for testing. Unfortunately, most individuals with positive UAT testing either did not have sputum cultures obtained or cultures were obtained following LD treatment, reducing sensitivity. Of the 80 individuals with positive UAT testing (of whom 73 were diagnosed with pneumonia), 36% (n = 29) did not have a dedicated *Legionella* sputum culture ordered, while 33% (n = 26) had an order but the specimen was not collected or the order was subsequently discontinued. There were 31% (n = 25) that had an order with cultures sent for evaluation and 4% (n = 3) that had sputum positive both by culture and PCR (Table 1).

**Table 1.** Demographics and clinical characteristics of patients with positive Legionella urine antigen testing stratified by insurance status across NYC H&H Hospitals during the NYC Legionella outbreak of 2025 Table 1 long description.

Variable	Overall N = 80	Medicare N = 26	Medicaid N = 33	Private insurance N = 18	Uninsured N = 3	*P*-value
**Race**						.3
White	1 (1.3%)	1 (3.8%)	0 (0%)	0 (0%)	0 (0%)	
Black or African American	68 (85%)	23 (88%)	27 (82%)	16 (89%)	2 (67%)	
Asian	2 (2.5%)	0 (0%)	1 (3.0%)	0 (0%)	1 (33%)	
Other	9 (11%)	2 (7.7%)	5 (15%)	2 (11%)	0 (0%)	
**Ethnicity**						.7
Hispanic or Latinx	9 (11%)	4 (15%)	4 (12%)	1 (5.6%)	0 (0%)	
Not Hispanic or Latinx	71 (89%)	22 (85%)	29 (88%)	17 (94%)	3 (100%)	
**Age, mean (SD)**	64 (16)	71 (15)	63 (17)	60 (12)	48 (7)	0.005
**Sex**						0.10
Male	43 (54%)	10 (38%)	21 (64%)	9 (50%)	3 (100%)	
Female	37 (46%)	16 (62%)	12 (36%)	9 (50%)	0 (0%)	
**Sputum**						0.034
Positive	3 (3.8%)	1 (3.8%)	1 (3.0%)	1 (5.6%)	0 (0%)	
Negative	22 (28%)	8 (31%)	4 (12%)	9 (50%)	1 (33%)	
Discontinued	26 (33%)	7 (27%)	17 (52%)	2 (11%)	0 (0%)	
No order	29 (36%)	10 (38%)	11 (33%)	6 (33%)	2 (67%)	
**Status**						>0.9
Alive	78 (97.5%)	25 (96%)	32 (97%)	18 (100%)	3 (100%)	
Deceased	2 (2.5%)	1 (3.8%)	1 (3.0%)	0 (0%)	0 (0%)	
**Transferred**						0.7
Harlem ED only	22 (28%)	7 (27%)	6 (18%)	7 (39%)	2 (67%)	
Harlem ED to Harlem admission	50 (63%)	17 (65%)	22 (67%)	10 (56%)	1 (33%)	
Harlem to Bellevue	6 (7.5%)	2 (7.7%)	3 (9.1%)	1 (5.6%)	0 (0%)	
Harlem to Lincoln	1 (1.3%)	0 (0%)	1 (3.0%)	0 (0%)	0 (0%)	
Left patient-directed	1 (1.3%)	0 (0%)	1 (3.0%)	0 (0%)	0 (0%)	

Descriptive statistics were used to summarize patient demographics and clinical characteristics. Continuous variables were expressed as means with standard deviations and compared across groups using the Kruskal-Wallis rank sum test, while categorical variables were expressed as frequencies and percentages (n, %). Fischer’s exact test was used to evaluate differences in categorical outcomes, including insurance status. Significance was defined as *p* < 0.05. Analyses were performed using R version 4.3.2.

## Focusing on equitable care for patients with *Legionella* infection

The DOH case definition included those who lived or visited the affected zip codes and met the Council of State Territorial Epidemiologists (CSTE) definition for LD.^14^ Among the 118 LD cases in the outbreak, 80 were from the affected Harlem zip codes and tested positive for *Legionella* on UAT across NYC H+H sites between July 21 and August 29, 2025. Demographics and clinical characteristics of these patients are summarized in Table 1.

Of the 80 individuals within the H+H Health system linked to the LD outbreak with positive UAT results, 85% (n = 68) identified as Black or African American and 54% (n = 43) were assigned male sex at birth. The average age (SD) was 64 (16), with 41% (n = 33) of this population insured by Medicaid, 33% (n = 26) by Medicare, 23% (n = 18) by private insurance and 4% (n = 3) uninsured. Patients insured by Medicaid had a higher proportion of sputum cultures that were discontinued, reflecting a potential operational gap in care to monitor and close in future outbreaks (Table 1). A summary of sputum test status by race and ethnicity is included in Table 2. Notably, patients who were identified as Hispanic/Latinx or Black in the EHR had higher rates of no order or order discontinued sputum cultures (89% and 69%, respectively), compared to non-Hispanic or White and Asian counterparts (66%, 0%, and 50%, respectively).

**Table 2.** Sputum test status by race and ethnicity Table 2 long description.

Variable	Positive N = 3	Negative N = 22	No order N = 29	Discontinued N = 26	Overall N = 80
**Race**					
White	0 (0%)	1 (100%)	0 (0%)	0 (0%)	1 (100%)
Black or African American	2 (2.9%)	19 (27.9%)	27 (39.7%)	20 (29.4%)	68 (100%)
Asian	0 (0%)	1 (50%)	0 (0%)	1 (50%)	2 (100%)
Other	1 (11.1%)	1 (11.1%)	2 (22.2%)	5 (55.6%)	9 (100%)
**Ethnicity**					
Hispanic or Latinx	0 (0%)	1 (11.1%)	2 (22.2%)	6 (66.7%)	9 (100%)
Not Hispanic or Latinx	3 (4.2%)	21 (29.6%)	27 (38%)	20 (28.2%)	71 (100%)

While 85% of patients linked to the 2025 LD outbreak were Black, 64% of the community in Central Harlem identifies as Black, compared with 21% in the overall NYC population.^15^ Furthermore, of the LD outbreak cohort from the affected zip codes, 41% were on Medicaid, while Central Harlem has comparable rates of its population on Medicaid at about 34%. Importantly, 6% of adults living in Central Harlem are uninsured, and our cohort had comparable uninsured rates (4%).^15^ Notably, Central Harlem has an elevated poverty rate compared to the overall NYCwide poverty rate (29% vs 18%).^16^ Tracking data allowed an ongoing assessment and evaluation of potential structural inequities in LD outbreak response that could serve as areas of future intervention.

Given the likelihood of ongoing transmission and to increase access to care for the Harlem community, patients were transferred from NYC H+H/Harlem to other H+H hospitals as part of a systemwide surge response, reflecting coordinated level loading of cases to avoid overwhelming any one hospital and to address geographic inequities given the disproportionate LD burden in Harlem. The NYC H+H/Bellevue intensive care unit and Internal Medicine leadership held daily clinical coordination calls with their counterparts at other H+H hospitals to determine each hospital’s patient care capacity and make daily decisions on patient transfers. Nursing leadership were key partners in load balancing, particularly addressing concerns around staffing and clinical handoffs, and ensuring seamless bed flow during transfers. H+H/Harlem medical teams identified patients with suspected or confirmed LD for transfer, including those with acute needs such as extracorporeal membrane oxygenation evaluation. Overall, this approach allowed for H+H/Harlem to better provide care for patients with suspected or confirmed Legionella presenting to their ED. In total, eight patients with any positive Legionella testing were transferred from NYC H+H/Harlem to NYC H+H/Bellevue during the period of the outbreak. Only one of these individuals had LD unrelated to the outbreak.

Formal ID consultation was sought for patients with severe disease or underlying immunocompromising conditions. At NYC H+H/Bellevue, three such patients with severe manifestations were formally evaluated by ID consult: one with severe acute renal injury due to LD-related rhabdomyolysis; another with anuric renal failure necessitating hemodialysis as well as significant encephalopathy with focal neurologic deficits; and the third with hypoxemic respiratory failure requiring intubation and severe, mixed shock requiring multiple pressors. Two out of those three patients required intensive care. No in-hospital deaths occurred at Bellevue and all patients received at least 7 days of either levofloxacin or azithromycin, or combination therapy, with more severe cases receiving up to 21 days of antibiotics. Cases seen on the ID consult service were reviewed with IP&C and information used to educate clinicians on variable clinical manifestations that should prompt high suspicion for LD and management of severe disease. In turn, ID clinician communication with families ensured those in the community potentially exposed to *Legionella* were informed about LD and could monitor for symptoms.

## Discussion

This commentary highlights key aspects of a how a safety-net hospital system coordinated with the NYC Health Department on its response to the 2025 NYC LD outbreak, with collaboration on early cluster detection, level loading of cases within a large public health system, and use of hospital surveillance systems to optimize outbreak investigation. The daily clinical coordination calls between NYC H+H/Bellevue and other H+H hospitals and communication through leadership of existing Clinical Councils allowed for widespread dissemination of information regarding LD evaluation and treatment to frontline providers. The surge in patients presenting to NYC H+H/Harlem led to coordinated transfers of patients to NYC H+H/Bellevue, direct involvement of ID clinicians in several complex cases, and increased local and systems capacity to treat patients from Central Harlem who were predominantly older, Black, insured by Medicaid, and facing higher poverty rates than the NYC average. The relationships between ID consultants, IP&C, and the NYC Health Department allowed for optimized patient care and outbreak investigation and mitigation.

NYC H+H/Bellevue’s IP&C department and the NYC Health Department prioritized a spatial epidemiological approach, screening all individuals from affected zip codes admitted with a diagnostic code of community-acquired pneumonia, to prioritize equitable access to care for H+H patients living in Harlem. This screening, pursued through EHR surveillance tools, paired with real-time communication with frontline clinicians, was not in place at the initiation of the outbreak and could be important for future outbreaks to ensure an equitable approach is part of the response.

Obtaining respiratory specimens for *Legionella* culture proved a persistent challenge, primarily due to early empiric antibiotic use and inherently poor growth of *Legionella* on culture, as well as other presumed factors, such as patients’ inability to produce sputum in non-severe cases. For patients presenting with critical illness, the focus was on initiating appropriate antibiotics; while clinicians understood the importance of diagnostic testing, cultures might have been delayed in these situations. Positive cultures are required for susceptibility testing and WGS for outbreak evaluation. The relatively small number of culture results limited early analyses to connect diagnosed cases with source cooling towers, highlighting the need for improved methods of pathogen detection earlier in outbreaks. Notably, only 32% of the H+H population with positive UAT testing had sputum cultures sent. Our analyses by insurance status and race/ethnicity shows there are gaps in equitable care to close for patients on Medicaid and Black and Latino patients, who had a higher percentage of sputum culture specimen orders either not sent or discontinued. Hospital systems can take a similar data-driven approach to identify gaps in outbreak response.

Future directions include improving coordination with clinical teams, such as respiratory therapists, who could be tasked with collecting induced sputum from all patients with suspected LD during an outbreak.^17^ Furthermore, an analysis examining outcomes in *Legionella* outbreaks by zip codes, and characterizing potential contextual factors such as distance from water tower locations, maintenance of such water towers, and *Legionella* testing discontinuation procedures could be another approach to explore potential inequities.

Health efforts to reduce further outbreaks due to *Legionella* should include maintaining access to health insurance, ongoing education for previously impacted communities on LD symptom recognition and when to present to care, monitoring and enforcement of cooling tower maintenance, and innovative public health monitoring systems that identify and track cases. Historically, Black communities have experienced disproportionate burden of infection during LD outbreaks. Multiple aspects contribute to these disparities. Cooling tower maintenance, which is the responsibility of the building owner, is a major consideration and may be a greater problem in lower income communities.^18^ Furthermore, racial and ethnic minorities continue to encounter disproportionate structural barriers in healthcare access, and community-level inequities such as inadequate housing infrastructure.^3^ In Central Harlem, 67% of tenant-occupied residences are reported to have one or more health-related maintenance concerns, such as water leaks.^15^ Individuals living in Central Harlem also suffer higher rates of avoidable hospitalizations when compared to the NYC population, reflective of poor access to primary care and correspondingly less management of comorbidities that could be risk factors for LD.^15^ Overall, the 2025 LD outbreak demonstrates the critical need to understand and respond to racial and socioeconomic disparities in LD and severe outcomes that may result as part of outbreak response.

## Conclusion

Here, we describe lessons learned from the outbreak response of ID physicians, IP&C practitioners and the NYC Health Department to the 2025 LD outbreak. The robust and efficient response by the NYC Health Department was critical to early identification of the outbreak source. The outbreak was contained within several weeks and declared over by the end of August 2025^1^. Effective, strong communication and partnership across hospital and health department systems was critical in understanding the extent of the outbreak and allowed for enhanced citywide case-finding and coordination of care, while the partnership between the NYC Health Department and the NYC H+H IP&C department allowed for improved outbreak surveillance and control. The importance of public health funding and infrastructure to maintain and expand these partnerships remains crucial in the prevention and control of future LD outbreaks.

## Data Availability

Data is not publicly available but is available upon reasonable request to the corresponding author.

## Figure 1. Long description

Panel A: A detailed map of New York City focusing on the Bronx area. The map shows Legionnaires disease case rates per 100,000 population, with different shades of blue indicating varying rates. Red and green dots represent source cooling towers and other cooling towers, respectively. ZIP code boundaries are outlined, and an environmental investigation area is marked with a buffer zone. Key locations such as Hamilton Heights, Concourse Village, and Mott Haven are labeled. Panel B: An inset map of New York City highlighting the specific area in the Bronx that is the focus of the detailed map.

Navigate back to Figure 1..

## Table 1. Long description

A table comparing demographics and clinical characteristics of patients with positive Legionella urine antigen testing stratified by insurance status across NYC H&H Hospitals. The table has 11 rows and 6 columns. Column headers are Variable, Overall N 80, Medicare N 26, Medicaid N 33, Private insurance N 18, Uninsured N 3, and P-value. Row labels include Race, Ethnicity, Age, mean (SD), Sex, Sputum, Status, and Transferred. Each row provides data for each insurance status category and overall. For example, under Race, 85 percent of overall patients are Black or African American, with similar percentages across insurance types. Under Ethnicity, 89 percent of overall patients are Not Hispanic or Latinx. The Age row shows the mean age and standard deviation for each group. The Sex row indicates the percentage of male and female patients. The Sputum row categorizes patients as Positive, Negative, Discontinued, or No order. The Status row shows the percentage of patients Alive or Deceased. The Transferred row details patient transfers between different hospital locations. Notable trends include a higher percentage of Black or African American patients and a majority of patients being Not Hispanic or Latinx. The mean age varies significantly across insurance types, with the uninsured group being the youngest. The table also highlights the distribution of sputum test results and patient status.

Navigate back to Table 1..

## Table 2. Long description

The table presents data on sputum test status by race and ethnicity for 80 individuals linked to an LD outbreak. It has 10 rows and 6 columns. The columns are labeled as Variable, Positive N = 3, Negative N = 22, No order N = 29, Discontinued N = 26, and Overall N = 80. The rows are labeled as Race and Ethnicity, with subcategories under Race including White, Black or African American, Asian, and Other, and subcategories under Ethnicity including Hispanic or Latinx and Not Hispanic or Latinx. Row 1: Race, White, 0 (0 percent), 1 (100 percent), 0 (0 percent), 0 (0 percent), 1 (100 percent). Row 2: Race, Black or African American, 2 (2.9 percent), 19 (27.9 percent), 27 (39.7 percent), 20 (29.4 percent), 68 (100 percent). Row 3: Race, Asian, 0 (0 percent), 1 (50 percent), 0 (0 percent), 1 (50 percent), 2 (100 percent). Row 4: Race, Other, 1 (11.1 percent), 1 (11.1 percent), 2 (22.2 percent), 5 (55.6 percent), 9 (100 percent). Row 5: Ethnicity, Hispanic or Latinx, 0 (0 percent), 1 (11.1 percent), 2 (22.2 percent), 6 (66.7 percent), 9 (100 percent). Row 6: Ethnicity, Not Hispanic or Latinx, 3 (4.2 percent), 21 (29.6 percent), 27 (38 percent), 20 (28.2 percent), 71 (100 percent).

Navigate back to Table 2..
